# Clinically Interpretable Radiomics-Based Prediction of Histopathologic Response to Neoadjuvant Chemotherapy in High-Grade Serous Ovarian Carcinoma

**DOI:** 10.3389/fonc.2022.868265

**Published:** 2022-06-16

**Authors:** Leonardo Rundo, Lucian Beer, Lorena Escudero Sanchez, Mireia Crispin-Ortuzar, Marika Reinius, Cathal McCague, Hilal Sahin, Vlad Bura, Roxana Pintican, Marta Zerunian, Stephan Ursprung, Iris Allajbeu, Helen Addley, Paula Martin-Gonzalez, Thomas Buddenkotte, Naveena Singh, Anju Sahdev, Ionut-Gabriel Funingana, Mercedes Jimenez-Linan, Florian Markowetz, James D. Brenton, Evis Sala, Ramona Woitek

**Affiliations:** ^1^ Department of Radiology, Cambridge, United Kingdom; ^2^ Cancer Research UK Cambridge Centre, University of Cambridge, Cambridge, United Kingdom; ^3^ Department of Biomedical Imaging and Image-Guided Therapy, Medical University of Vienna, Vienna, Austria; ^4^ Cancer Research UK Cambridge Institute, University of Cambridge, Cambridge, United Kingdom; ^5^ Department of Oncology, University of Cambridge, Cambridge, United Kingdom; ^6^ Cambridge University Hospitals NHS Foundation Trust, Cambridge, United Kingdom; ^7^ Department of Radiology, Tepecik Training and Research Hospital, Izmir, Turkey; ^8^ Department of Radiology and Medical Imaging, County Clinical Emergency Hospital, Cluj-Napoca, Romania; ^9^ Department of Radiology, Iuliu Hațieganu University of Medicine and Pharmacy, Cluj-Napoca, Romania; ^10^ Department of Surgical and Medical Sciences and Translational Medicine, Sapienza University of Rome—Sant’Andrea University Hospital, Rome, Italy; ^11^ Department of Applied Mathematics and Theoretical Physics, University of Cambridge, Cambridge, United Kingdom; ^12^ Department of Clinical Pathology, Barts Health NHS Trust, London, United Kingdom; ^13^ Department of Radiology, Barts Health NHS Trust, London, United Kingdom

**Keywords:** ovarian cancer, radiomics, computed tomography, chemotherapy response score, neoadjuvant chemotherapy

## Abstract

**Background:**

Pathological response to neoadjuvant treatment for patients with high-grade serous ovarian carcinoma (HGSOC) is assessed using the chemotherapy response score (CRS) for omental tumor deposits. The main limitation of CRS is that it requires surgical sampling after initial neoadjuvant chemotherapy (NACT) treatment. Earlier and non-invasive response predictors could improve patient stratification. We developed computed tomography (CT) radiomic measures to predict neoadjuvant response before NACT using CRS as a gold standard.

**Methods:**

Omental CT-based radiomics models, yielding a simplified fully interpretable radiomic signature, were developed using Elastic Net logistic regression and compared to predictions based on omental tumor volume alone. Models were developed on a single institution cohort of neoadjuvant-treated HGSOC (*n* = 61; 41% complete response to NCT) and tested on an external test cohort (*n* = 48; 21% complete response).

**Results:**

The performance of the comprehensive radiomics models and the fully interpretable radiomics model was significantly higher than volume-based predictions of response in both the discovery and external test sets when assessed using G-mean (geometric mean of sensitivity and specificity) and NPV, indicating high generalizability and reliability in identifying non-responders when using radiomics. The performance of a fully interpretable model was similar to that of comprehensive radiomics models.

**Conclusions:**

CT-based radiomics allows for predicting response to NACT in a timely manner and without the need for abdominal surgery. Adding pre-NACT radiomics to volumetry improved model performance for predictions of response to NACT in HGSOC and was robust to external testing. A radiomic signature based on five robust predictive features provides improved clinical interpretability and may thus facilitate clinical acceptance and application.

## 1 Introduction

Over the past 15 years, there has been a dramatic rise in the use of neoadjuvant chemotherapy (NACT) for advanced high-grade serous ovarian cancer (HGSOC) where patients receive 3–4 cycles of carboplatin and paclitaxel before delayed primary surgery (DPS) when immediate primary surgery (IPS) is not feasible (1–3). NACT is used as frontline therapy for >60% of the HGSOC patients in the UK and for >45% in the US (4, 5). Early assessment of treatment response following NACT provides predictive information for the effectiveness of DPS and survival (5). Pathological complete response after NACT is the strongest predictor of outcome in many epithelial cancers and can be a robust surrogate biomarker for clinical trials (6–8). However, assessing pathological response in HGSOC is complex because of multisite disease with heterogeneous tumor microenvironments (9, 10), and diverse clonal populations (11, 12). The three-tier chemotherapy response score (CRS) assesses histopathological response in omental tumor deposits to stratify patients into three response groups: none or minimal (CRS1), partial (CRS2), or complete response (CRS3). A meta-analysis of 877 patients showed that complete response (CRS3) is associated with prolonged progression-free survival (PFS) and overall survival (OS) (13). CRS is therefore the best-validated candidate for use as an early surrogate biomarker of response in HGSOC (13).

The main limitation of CRS is the requirement for omental surgery. Consequently, CRS may be difficult to apply for all patients receiving NACT whereas computed tomography (CT) is routinely used to assess response and can predict PFS (14). While the CRS assesses response on a microscopic level, CT detects changes on a meso- to macroscopic level and could provide complementary information. There are still significant challenges to develop sensitive imaging biomarkers of response as previous studies have not shown positive associations between RECIST 1.1 response and CRS (15). Radiomics provides advanced quantitative analyses of radiological images (16–18) and is predictive of treatment response in HGSOC and other cancers (19, 20). We therefore developed methods to predict clinical response to NACT by combining radiomics with omental tumor volumetry and using CRS as the gold standard.

## 2 Materials and Methods

**Figure 1:**
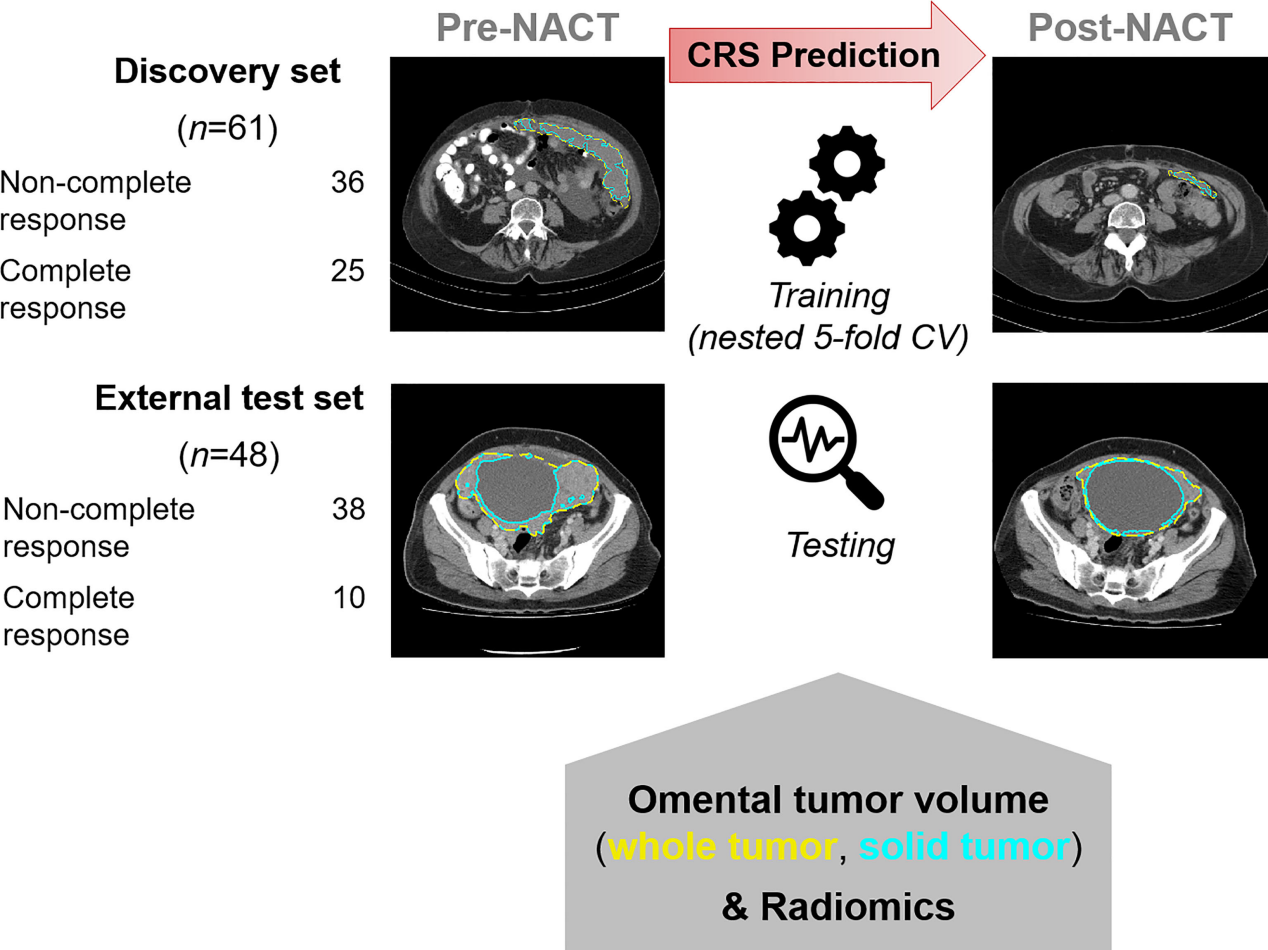
Overall design of the study for identifying radiomic predictors of CRS-confirmed response. Pre- and post-NACT CT images were analyzed. CRS classification is tabulated.

This is a retrospective analysis of prospectively collected data from the Cambridge University Hospitals NHS Foundation Trust (Cambridge, UK) and the Barts Health NHS Trust (London, UK). This study was approved by our institutional review boards (REC reference numbers 08/H0306/61 and IRAS reference number 243824). Written informed consent was obtained from all participants. Clinical and outcome data from patients at the Barts Health NHS Trust were reported in a previous publication, in which no imaging data were included (21). **Figure 1** shows the overall design of the study.

### 2.1 Materials

#### 2.1.1 Study Participants

Research participants were consecutively and prospectively recruited from the Cambridge University Hospital between 2009 and 2017 (discovery cohort), and from the Barts Health NHS Trust, between October 2009 and October 2014 (external test cohort). The inclusion criteria were patients aged 18 years or older, histological diagnosis of HGSOC, NACT before DPS, pre- and post-NACT contrast-enhanced CT of the abdomen and pelvis, pre-NACT omental tumor deposits >3 cm³, and CRS assessment based on surgical specimens obtained from DPS. In both centers, the recommendation for NACT and DPS instead of IPS was based on the selection criteria published by the ESMO–ESGO Ovarian Cancer Consensus Conference Working Group (22, 23). After careful evaluation of patients before surgery, a management plan was defined in a multidisciplinary setting. If resection of all macroscopic disease was not deemed obtainable based on pre-operative staging with acceptable operative morbidity, NACT and DPS were recommended. Both disease- and patient-specific factors (such as coexisting illnesses, age, and performance status) were considered in the decision-making process.

#### 2.1.2 Clinical Data

Patients’ medical records at the Cambridge University Hospitals were reviewed by a medical oncology resident with 2 years of specialty training (MR) under the supervision of a board-certified medical oncologist (JB) with >20 years of experience. Patients’ medical records at the Barts Health NHS Trust were reviewed by members of the clinical care team at the local center. Demographic data are shown in **Supplementary Table S1**.

#### 2.1.3 Histopathologic Analysis

The three-tier CRS was assigned by board-certified pathologists with subspecialty training in gynecological oncology at both centers using previously published criteria (9, 21). Briefly, the section of the omentum showing the most residual viable tumor was assigned a score based on the response of the omental tumor to chemotherapy: score 1 = abundant tumor with no or minimal perceptible response to chemotherapy; score 2 = significant amount of viable tumor present, showing readily appreciable fibro-inflammatory response secondary to treatment; score 3 = complete or near-complete response with no tumor or minimal irregularly scattered tumor nests (none > 2 mm).

The three-tier CRS outcomes were dichotomized into non-complete response (CRS1-2) and complete response (CRS3) and used for all model fitting analyses.

#### 2.1.4 CT Acquisition

CT acquisition parameters are given in **Supplementary Table S4**. Spatial voxel resolution, kilovoltage peak (kVp), and reconstruction kernel values were variable, as CT scanners from different institutions with different vendors and models were used. The axial contrast-enhanced images reconstructed with a soft tissue kernel were analyzed.

#### 2.1.5 CT-Based Tumor Segmentation

Omental tumor deposits from patients included at the Cambridge University Hospitals were manually 3D segmented by a board-certified radiologist (RW) with 10 years of experience in radiology. Omental tumor deposits from patients included at Barts Health NHS Trust were initially manually segmented by a radiology resident (VB, CM, LB, RP, and MZ) with 1 to 6 years of experience in radiology and reviewed by one of the two board-certified radiologists (RW and ES). The segmentation on both datasets was performed using the Microsoft Radiomics App v1.0.28434.1 (project InnerEye; Microsoft, Redmond, WA, USA; https://www.microsoft.com/en-us/research/project/medical-image-analysis).

We applied an automated tissue-specific sub-segmentation method previously developed (24) to the manual whole tumor segmentation. This sub-segmentation allowed us to reliably exclude hypodense (i.e., fatty or cystic/necrotic) and hyperdense (i.e., calcified) components from the intermediately dense (i.e., soft tissue) portions of the omental tumor.

**Figure 1** shows two examples of omental lesions delineated on the CT images for both datasets and time points (the zoomed version of the CT images is provided in **Supplementary Figure S1**).

#### 2.1.6 RECIST 1.1 Assessment

CT scans for all patients were assessed according to the RECIST 1.1 response criteria (25) by a board-certified radiologist.

### 2.2 Methods

#### 2.2.1 Volumetric Analyses

First, the volumetric measurements of the omental lesions pre- and post-NACT, as well as the percentage change between the two time points were calculated.

A Wilcoxon rank-sum test (Mann–Whitney *U* test) was used to assess the statistical differences between responders and non-responders. For predictive modeling, we employed a univariate logistic regression between the volumetric measurements (for both whole tumors and soft tissue components) and the dichotomized CRS as the response variable.

#### 2.2.2 Radiomics Analyses

**Figure 2:**
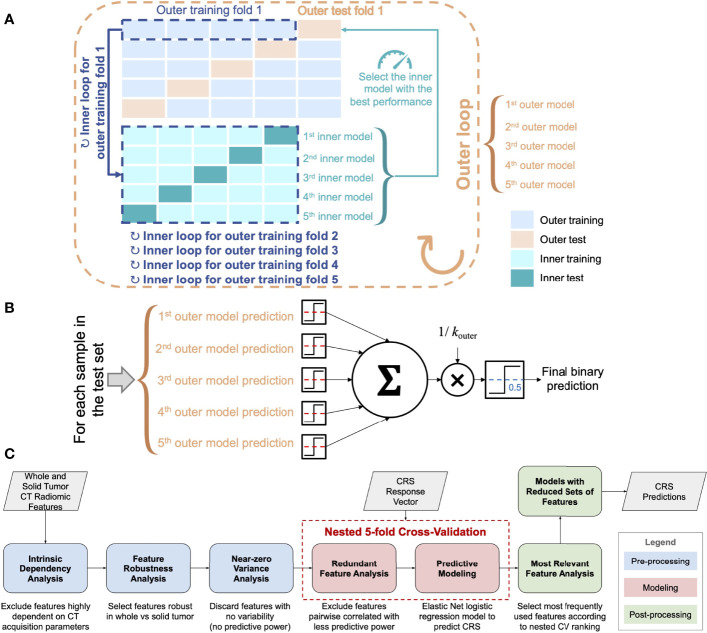
**(A)** Scheme of the nested *k*-fold cross-validation (for *k*_outer_ = 5 and *k*_inner_ = 5). The nested fitting procedure was repeated 100 times with different random permutations of the discovery dataset. **(B)** Majority voting for the ensemble of classifiers used for testing on the external test cohort (the dashed red lines denote the decision thresholds optimized according to the inner CV loop). **(C)** Workflow of the radiomics pipeline for CRS prediction.

The processing and analysis steps are outlined in **Figure 2C** and described in the following sections.

##### 2.2.2.1 Radiomic Feature Extraction, Calibration, and Pre-Processing

The analyzed features were extracted using PyRadiomics version 2.0 (26) in Python 3.7.5 Along with 3D shape-based features, six feature classes were extracted: (1) first-order intensity histogram statistics, (2) Gray-Level Co-occurrence Matrix (GLCM) features (27, 28), (3) Gray-Level Run Length Matrix (GLRLM) (29), (4) Gray-Level Size Zone Matrix (GLSZM) (30), (5) Gray-Level Dependence Matrix (GLDM) (31), and (6) Neighboring Gray Tone Difference Matrix (NGTDM) (32). All the radiomic features are listed in **Table S5**.

3D feature computation used a resegmentation (i.e., the voxels outside a specified range are removed from the mask prior to texture feature calculation) in [−100, …, 400] Hounsfield units (HU) and the original voxel sizes. The quantization settings (33) were derived using the Freedman-Diaconis rule, an extension of Scott’s rule to non-Gaussian distributions (34, 35).

The details on feature calibration and pre-processing are provided in **Supplementary Materials**.

##### 2.2.2.2 Predictive Modeling

Prior to the predictive modeling phase, we evaluated the redundancy among all features and removed highly correlated features (36). We used the Spearman’s correlation coefficient *ρ* for pairwise feature comparison. In the case of *ρ* ≥ 0.90, the feature with the highest predictive power was selected. This selection relied upon a univariate logistic regression for predicting the dichotomized CRS and removing the feature that achieved the lowest area under the receiver operating characteristic (AUC). Since this redundant feature analysis needs the CRS response variable to select the most predictive feature, to keep each outer test fold completely unseen, the procedure was embedded in the inner loop of the nested *k*-fold CV.

The predictive modeling made use of the Elastic Net regularization for logistic regression with the dichotomized CRS as the response variable (37). The predictive models were trained and tested on the development cohort *via* a nested *k*-fold CV procedure. In particular, in the inner loop, the *k*-fold CV aimed at minimizing the λ-penalized deviance, thus optimizing the value of𝜆 the shrinkage parameter. The analyzed features were standardized using a *z*-score transformation.

##### 2.2.2.3 Post-Processing and Relevant Feature Analysis

Relying upon the achieved predictive model results, the most relevant features were analyzed in terms of the occurrences (i.e., when a feature coefficient is non-zero). Therefore, Elastic Net was fitted on this reduced subset of features using the same nested *k*-fold CV scheme and settings (also in terms of data partitioning).

#### 2.2.3 Statistical and Computational Analysis

Statistical and computational analyses were performed using MatLab^®^ R2019b (64-bit version) environment (The MathWorks, Natick, MA, USA) and SPSS (version 26; IBM, USA).

Continuous variables were reported as mean and standard deviation (SD) given normally distributed data or median and IQR when skewed, while categorical variables were reported as number and percentage of patients with the specific characteristics.

One-way analysis of variance was used for group comparisons of continuous variables, when applicable. Otherwise, a Kruskal–Wallis test was applied. Group comparisons of categorical variables were performed using the *χ* ^2^ or Fisher exact test, as appropriate. A *p*-value ≤ 0.05 was considered as statistically significant.

For distribution comparisons, the non-parametric Wilcoxon rank-sum test (Mann–Whitney *U* test) was used, using a significance level of 0.05. In the case of multiple comparisons, the *p*-values were adjusted using the Bonferroni–Holm method.

For correlations between summed longest diameters (SLDs) according to RECIST 1.1 with dichotomized CRS, Spearman’s correlation coefficients were computed.

The used evaluation metrics were the AUC and classification accuracy, along with Positive Predictive Value (PPV) and Negative Predictive Value (NPV) to better investigate true positive and true negative results, respectively (38). We also considered the sensitivity and specificity, as well as the geometric mean (G-mean) defined as $\sqrt{\text{Sensitivity}\times \text{Specificity}}$ . For comparing matched samples, the non-parametric Wilcoxon signed-rank test on paired samples was used, using a significance level of 0.05.

#### 2.2.4 Training and Testing Methodology

For both volumetric and radiomics analyses, the predictive models were fitted on the discovery cohort in nested 5-fold cross-validation (CV) as shown in **Figure 2A** and the **Supplementary Materials**.

For the test on the external cohort, an ensemble of the 5 cross-validated Elastic Net models fitted on the discovery cohort was used (**Figure 2B**). We used majority voting methods based on the single predictions that employ the optimized decision thresholds.

## 3 Results

### 3.1 Characteristics of Patient Cohorts

**Figure 1** summarizes the study design. The study cohort included a training set of 61 patients and an independent, external test set of 48 patients receiving NACT for HGSOC. **Figure S2** provides a REMARK diagram for case identification and **Table S1** summarizes clinical details for the cohorts. Patients were followed for a median of 37 (IQR 26–48) and 35 (IQR 24–44) months in the training and external test cohorts, respectively. The training and test cohorts had different proportions for pathological complete response (CRS3). Complete response (CRS3) was observed in 25/61 (41%) of patients in the training set and 10/48 (21%) patients in the external test set. Non-complete response (CRS1–2) was by trend a risk factor for disease progression and death in both groups (**Tables S2, S3**). Measurements of the summed longest diameter of target lesions according to RECIST 1.1 were not significantly correlated with the dichotomized CRS in both cohorts [discovery cohort: pre-NACT *⍴* = –0.026 (*p* = 0.845), post-NACT *⍴* = –0.156 (*p*=0.232); external test cohort: pre-NACT *⍴* = 0.124 (*p* = 0.401), post-NACT *⍴* = –0.013 (*p* = 0.930).

### 3.2 Smaller Omental Tumor Volume Is Associated With Complete Response

**Figure 3:**
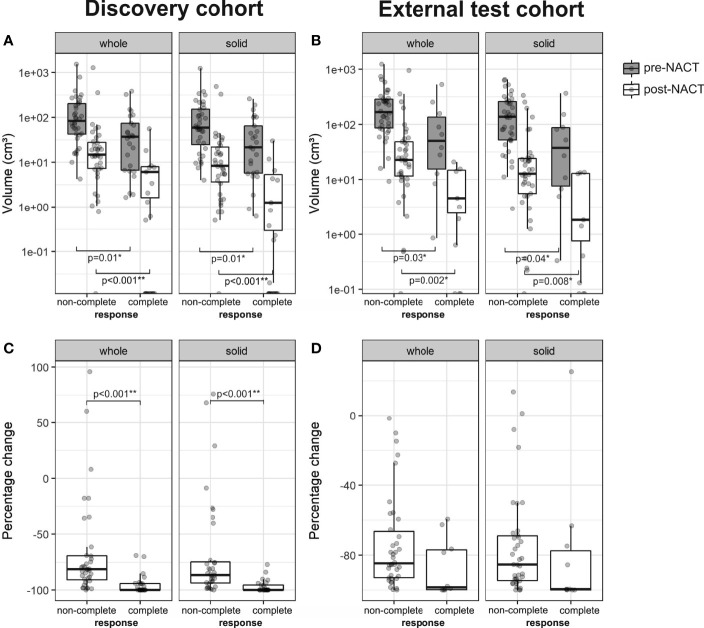
Boxplots of the whole tumor and solid tumor volume in patients with non-complete (CRS1-2) and complete response (CRS3) from the **(A)** discovery (*n* = 61, non-complete response = 36, complete response = 25) and **(B)** external test cohorts (*n* = 48, non-complete response = 38, complete response = 10). Percentage change of whole tumor and solid tumor volume is shown in **(C)** for the discovery cohort and in **(D)** for the external test cohort. For pre- and post-NACT volumes, a logarithmic scale was used on the *y*-axis.

We first assessed whether patients with complete response (CRS3) had significantly different omental tumor volumes (on pre- and post-NACT CT scans) when compared to patients with non-complete response (CRS1–2). In the discovery cohort, patients with complete response had significantly smaller omental tumor volumes, both pre-NACT (median 36.9 cm³ vs. 84.6 cm³; *p* = 0.01) and post-NACT (median 0.00 cm³ vs. 14.6 cm³; *p* ≪ 0.001), and also showed a larger negative percentage change (median: −100.0% vs. −81.5%; *p* ≪ 0.001) (**Figures 3A, C**). In the external test set, patients with complete response also had significantly smaller pre- and post-NACT omental tumor volumes (median 51.9 cm³ vs. 166.7 cm³; *p* = 0.03; 2.5 cm³ vs. 22.7 cm³; *p* = 0.002, respectively), but the percentage change was not significantly different (median –98.5% vs. −84.7%; *p* = 0.07) compared to patients with non-complete response (**Figures 3B, D**).

Ovarian carcinoma metastases are mesoscopically heterogeneous. CT appearances include solid/soft tissue (intermediately dense) tumor as well as cystic/necrotic (hypodense) and calcified (hyperdense) components. Different components in the same metastasis may show differential response to chemotherapy. We identified solid/soft tissue and cystic/necrotic tumor components using an automated sub-segmentation method (22) and evaluated the volume of solid tumor components at the two time points pre- and post-NACT. Results for the volume of solid tumor components are similar to those of whole tumor volume for patients with complete and non-complete response (**Figure 3**). Therefore, further analyses focused on solid tumor volume alone.

### 3.3 Omental Tumor Volume Predicts Complete Response and the Prediction Improves With the Addition of Radiomics

**Figure 4:**
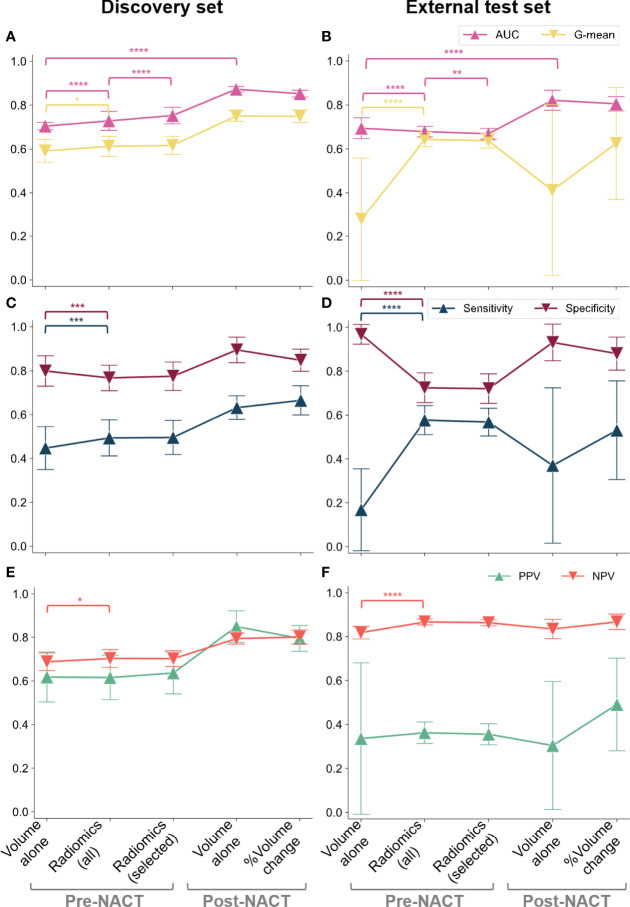
CRS classification results in terms of AUC and G-mean (first row), along with sensitivity and specificity (second row) and PPV and NPV (third row): **(A, C, E)** discovery cohort; **(B, D, F)** external test set. We considered the pre-NACT volumetric model and radiomic models fitted on either all the preprocessed features (robust and non-redundant) or only on the most frequently selected (i.e., relevant) features along with omental tumor volume. The variability across 100 repetitions was considered. The dots and error bars denote the average value and the standard deviation, respectively. Brackets denote statistical significance of particular interest using a Wilcoxon rank-sum test. Notation: *p < 0.05, **p < 0.01, ***p < 0.001, ****p ≪ 0.0001.

We next investigated whether complete response could be predicted from pre- and post-NACT omental tumor volume and the percentage change in omental tumor volumes. Using univariable logistic regression, smaller values for the omental tumor volume measured pre- and post-NACT, as well as a larger percentage change in response to NACT, were correlated with complete response in both the discovery and the external test sets. We used nested 5-fold CV of the discovery set (**Figure 2A**) to estimate the AUC for sensitivity and specificity of complete response prediction. Model performance metrics are shown in **Figure 4**. The AUC ranged between 0.68 and 0.87 using either the pre- or post-NACT omental tumor volumes from both the discovery and the external test sets (**Figure 4**). The post-NACT volumetric data significantly improved model AUC compared to pre-NACT volumetry in both cohorts (**Figures 4A, B**) (*p* ≪ 0.0001 in both cases). Excluding cystic/necrotic and calcified areas from the volumetric analysis and only taking into account solid tumor components did not improve the performance of the volume-based models (**Supplementary Figure S3**).

To evaluate if prediction of complete response could be improved by including radiomic analysis of pre-NACT omental tumors, we first pre-processed 107 potential radiomic features to identify a smaller robust and non-redundant set of 42 radiomic features, which included two measurements for volume (mesh volume and voxel volume).

In the discovery set, the pre-NACT radiomic models significantly improved the AUC compared to volumetric models (*p* ≪ 0.0001). Although this effect was not observed in the external test dataset where the volumetric model had the highest AUC (*p* ≪ 0.0001) (**Figures 4A, B**), the inclusion of radiomic data into the model significantly improved the NPV of our predictions compared to the volume-based model (*p* ≪ 0.0001), indicating increased reliability for the identification of non-responders at this early time point in the external test dataset (**Figures 4E, F**). Furthermore, the radiomics-based model strongly decreased the wide variability of PPV from using only volumetric data (SD reduced from 0.345 to 0.049) (**Figures 4E, F**). Similarly, a higher G-mean—which is a summary measure of the sensitivity and specificity of the test—was observed for the radiomics-based models compared to the volume-based model demonstrating more stable detection performance (*p* < 0.05 and *p* ≪ 0.0001 on the discovery and external test sets, respectively) (**Figures 4A, B**).

### 3.4 A Simplified and Interpretable Radiomics Model Maintains High Prediction Accuracy

Radiomics-based prediction models are frequently criticized for their lack of interpretability and explainability, particularly as models with a large number of radiomic features are not clinically meaningful. Identifying simplified models that have good prediction accuracy is essential to increase the acceptance of prediction models by clinicians and their incorporation into clinical care. Therefore, we aimed to create an additional simplified prediction model based on a subset of radiomics features (i.e., relevant features), which we identified from the 42 features that were most frequently selected for the 500 trained models fitted after the pre-processing steps (see **Figure S5**). The five features shown in **Table 1** were selected more than 300 times, and they were selected more often than the tumor volume that had moderate predictive power (**Figure S6**).

**Table 1:** List of the features selected and included in the radiomic signature. Mean values of the coefficients of the Elastic Net logistic regression (averaged over 500 model instances).

Feature group	Feature name	Description	Coefficient
Shape	Maximum 2D diameter (column)	The largest pairwise Euclidean distance between tumor surface mesh vertices in the coronal plane	–0.1815
	Least Axis Length	The smallest axis length of the ROI-enclosing ellipsoid	–0.241
	Elongation	Describes the relationship between the two largest principal components in the ROI shape	0.379
GLCM (gray-level co-occurrence matrix)	Inverse Difference Moment Normalized (IDMN), also denoted as homogeneity	Is a measure of the local homogeneity of an image (IDMN weights are the inverse of the contrast weights (decreasing exponentially from the diagonal i=j in the GLCM). It measures the smoothness (homogeneity) of the gray-level distribution of the image; it is (approximately) inversely correlated with contrast—if contrast is small, usually homogeneity or IDMN is large (39)	–0.7857
	Difference Entropy	A measure of the randomness/variability in neighborhood intensity value differences; measures the degree of disorder related to the gray-level difference distribution of the image. Entropy is (approximately) inversely correlated with uniformity; images with a larger number of gray levels have larger entropy (39)	–0.4252
Volume			–0.0458

The response variables were codified as 1 = CRS3, 0 = CRS1–2.

In addition to these five features, we included the whole omental tumor volume in our final simplified model because it has high clinical recognition. In the discovery cohort, this model achieved a significantly higher mean AUC than the models fitted on all 42 features (0.75 ± 0.04 and 0.73 ± 0.04, respectively; *p* ≪ 0.0001), and in the external test set, its AUC was significantly lower than that of the models based on 42 radiomics features (0.68 ± 0.03 and 0.69 ± 0.02, respectively; *p* = 0.0006) (**Figures 4A, B**). Accuracy was not significantly affected (*p* = 0.626).

Therefore, as expected, the simplified models that used only the most frequently selected features showed slightly lower generalization abilities on the external test dataset.

**Figure 4** summarizes the overall results for volumetric, radiomics-based, and simplified radiomics-based models, on both the discovery and external test sets, and demonstrates the relationship between model sensitivity, specificity, and AUC. In the discovery set, radiomics-based models showed higher AUC than the volume-based model (**Figure 4A**) while the opposite was observed in the external test set: the AUC of radiomics-based models was inferior to volume-based predictions (**Figure 4B**). Although these results may seem discouraging for the use of radiomics, further exploration of the datasets and sensitivity and specificity of the models indicates that this may be a result of a high class imbalance in the external test set. To explore this further, we calculated the geometric mean of the sensitivity and specificity as this can better define a centrality measure for the model evaluation in the case of imbalanced classification performance. Owing to the class imbalance, the sensitivity and specificity of the volume-based predictions reached extreme values on the external test set (**Figure 4D**) when compared to the discovery dataset (**Figure 4C**). This drastic change in sensitivity and specificity when moving to the external test set is a sign of poor generalizability of the volume-based model. Although these extreme values lead to a higher overall AUC for volume-based predictions compared to radiomics based models, it is high generalizability that is the aim of model development and, therefore, models achieving sensitivity and specificity in an external test set that are comparable to those achieved on the discovery set are preferable. Radiomics-based models (both models including all 42 radiomics and the simplified model) thus demonstrate the advantage of higher generalizability when compared to purely volume-based predictions. In addition, the simplified radiomics model has a significant clinical advantage in being fully interpretable and achieves sensitivity, specificity, and AUC values comparable to the full radiomics model.

### 3.5 Interpretation of the Radiomic Signature

The mean values of the coefficients of the Elastic Net logistic regression (averaged over 500 instances) for the radiomic model fitted on the selected features are shown in **Table 1**. The coefficient values showed that response was associated with omental lesions characterized by a more elongated shape with lower least axis length and maximum 2D diameter in the coronal plane, compared to non-responders. Also, response was associated with low homogeneity (low IDMN) and with low difference entropy [both are GLCM features capturing tumor heterogeneity (40)]; low homogeneity indicates high contrast within the tumor deposit of responders. Low entropy is a typical feature of a lesion exhibiting a low number of different gray levels. However, difference entropy is computed on the intensity difference between neighboring voxels, indicating that these differences were smaller (or, in other words, neighboring gray levels were more similar) in responders compared to non-responders. These results are confirmed by the boxplots depicted in **Supplementary Figures S7, S8**. To investigate the influence of non-solid/soft tissue components on the radiomic signature defined, **Table 1** also reports the Spearman’s correlation coefficient for each radiomic feature computed on whole and solid/soft tissue tumor component VOIs for both the discovery and external test sets. All the values of the Spearman’s correlation coefficient confirm a high correlation, especially for the shape-based features, thus showing that these radiomic features are not considerably affected by the non-solid/soft tissue components (e.g., hypodense or hyperdense regions potentially present within the tumor).

## 4 Discussion

NACT followed by DPS is an accepted alternative treatment for patients with advanced HGSOC where complete resection during IPS may not be achievable based on clinical and imaging findings at presentation. However, for patients with poor performance status and fitness for cytotoxic therapy, the decision whether to proceed with NACT (often with adjustments, such as dose reduction and/or single-agent therapy) in the hope of symptomatic improvement, or to opt for best supportive care (i.e., no oncological treatment) can be extremely challenging. In this scenario, an objective prediction of lack of response (which would imply toxicity without symptomatic improvement with NACT) could play an important role in informing discussions with patients and clinical decision-making. Although the majority of HGSOC patients respond to standard first-line therapy, it has been shown recently (5) that complete or partial response rates to first-line NACT are lower than previously thought. Future development of clinical trials of alternative neoadjuvant approaches for first-line non-responders is thus a key priority. Prediction tools like the ones shown here are therefore required to allow for confident prediction of lack of response at presentation in order to facilitate recruitment to such clinical trials. CRS criteria are validated to evaluate changes in omental tumor deposits on surgical specimens, which represents a shortcoming in incorporating CRS as a stratification tool for prospective clinical trials of novel neoadjuvant antineoplastic agents but could be overcome by imaging-based prediction tools of response to standard-of-care NACT.

Although omental tumor volumes pre- and post-NACT alone can predict CRS, we showed that predictions can be significantly improved with radiomics-based models. Only these models were robust enough to reduce standard deviation of performance metrics on highly unbalanced data as observed in our external test set and significantly improved the NPV of predictions allowing to reliably identify non-responders.

We developed a fully interpretable prediction model based on only six highly robust features marking a transition point from a black-box approach—using large numbers of uninterpretable radiomics features—to a more intuitive model that is limited to a smaller number of features but preserves generalizability and accuracy in its predictions. We found that features quantifying lesion size and shape were among the most relevant ones. These findings highlight that unidimensional tumor measurements in the axial plane (as performed in routine clinical practice and for RECIST 1.1) and even tumor volume measurements alone are insufficient to capture the most relevant size- and shape-related properties for predicting response. They disregard the two properties selected as highly robust and relevant: tumor extent in the supero-inferior direction and tumor elongation. Even manual assessment of these features on coronally reconstructed CT images could easily be performed by the reporting radiologist, which highlights that the results obtained from our study could possibly be immediately implemented in routine clinical image interpretation and reporting.

We investigated the use of tissue-specific sub-segmentation proposed in (22). To date, the majority of quantitative imaging studies disregard macroscopic tumor heterogeneity, even though solid tumor regions typically have high cellular density and could contribute more to adverse prognostic or predictive information than necrotic, cystic, or calcified regions (41). However, our results showed no significant changes between the AUC of logistic regression models for predicting CRS when compared to whole tumor volume. For this reason, and to ensure that the clinically interpretable criteria suggested by this paper could be used by any center with no further requirements, our results were obtained using the whole omental disease.

A CT-based radiomic prognostic vector associated with molecular features of ovarian cancer has been proposed for prognostication and patient stratification previously (42, 43). Radiomic–clinical nomograms have also shown prognostic and predictive power recently (44–47): Hong et al. used a combination of CT images and clinical features (47), whereas the work published by Wang et al. incorporated radiomics from hybrid ^18^F-FDG PET/CT together with clinical features (46) for prognostication. Li et al., on the other hand, used MRI-based radiomics to predict surgical outcome (45). To our knowledge, this is the first study predicting response to NACT in ovarian cancer as assessed by the gold standard CRS, and it is not taking into account ovarian lesions but analyzes the radiomics of omental disease. The omentum harbors a unique immune environment (48, 49), is the most common site of spread in ovarian cancer, and is the anatomic site where response to NACT is histopathologically assessed using the CRS as the gold standard making it an ideal anatomic site for radiomics analysis. This study has several limitations. First, the study was not powered to associate the CRS, omental tumor volumes, and the radiomic signatures with clinical endpoints, such as PFS or OS, which will be the aim for future multicentric studies. Second, the selection criteria for considering IPS *versus* NACT followed by DPS are not yet fully standardized across different centers. However, this reflects clinical practice and we have shown that, even between these heterogeneous study cohorts, the radiomic signature defined is generalizable and applicable. Third, although the CRS has previously been shown to have high inter-reader agreement (50), histopathological assessment bias cannot be ruled out in this setting where CRS assessments were made by different pathologists in different institutions. For future studies, consensus assessment by multiple assessors or centralized pathology review may be considered. We conclude that CT-based volumetric analysis of omental tumor deposits can predict CRS, and its predictive ability can be improved further by adding pre-NACT radiomics.

In conclusion, we show that pre- and post-NACT volumetry of omental deposits in HGSOC predicts CRS. These predictions were further improved by adding radiomics resulting in a fully interpretable radiomics model that also increased model generalizability, along with robustness, and could aid in identifying patients with predicted lack of response to first-line chemotherapy as possible candidates for trials of alternative neoadjuvant approaches.

## Data Availability Statement

The datasets presented in this article are not readily available due to ethical constraints. Requests to access the datasets should be directed to rw585@cam.ac.uk.

## Ethics Statement

The studies involving human participants were reviewed and approved by Institutional Review Boards of the Cambridge University Hospitals NHS Foundation Trust (Cambridge, UK) and the Barts Health NHS Trust (London, UK) (REC reference numbers 08/H0306/61 and IRAS reference number 243824). The patients/participants provided their written informed consent to participate in this study.

## Author Contributions

Conceptualization: LR, LB, LES, MC-O, NS, AS, JB, ES, and RW. Data curation: LR, LB, LES, MC-O, MR, CM, HS, VB, RP, MZ, HA, NS, AS, JDB, ES, and RW. Formal analysis: LR, LB, LES, MC-O, JB, ES, and RW. Funding acquisition: JB and ES. Investigation: LR, LB, LES, MC-O, MR, JB, ES, and RW. Methodology: LR, LB, LES, MC-O, NS, AS, JB, ES, and RW. Project administration: JB and ES. Resources: JB and ES. Software: LR, LB, LES, MC-O, NS, AS, JB, ES, and RW. Supervision: MC-O, JB, ES, and RW. Validation: LR, LB, LES, MC-O, MR, CM, HS, VB, RP, MZ, HA, NS, AS, JB, ES, and RW. Visualization: LR, LB, LES, MC-O, JB, ES, and RW. Writing—original draft: LR, LB, LES, MC-O, JB, ES, and RW. Writing—review and editing: LR, LES, MR, CM, HL, VB, RP, MZ, SU, IA, HA, PM-G, TB, NS, AS, I-GF, MJ-L, FM, JB, ES, and RW. All authors contributed to the article and approved the submitted version.

## Funding

This work was supported by The Mark Foundation for Cancer Research and Cancer Research UK Cambridge Centre [C9685/A25177], the Wellcome Trust Innovator Award, UK [215733/Z/19/Z], and the CRUK National Cancer Imaging Translational Accelerator (NCITA) [C42780/A27066] and Cancer Research UK grant 22905 [JB]. Additional support was also provided by the National Institute of Health Research (NIHR) Cambridge Biomedical Research Centre (BRC-1215-20014). The views expressed are those of the authors and not necessarily those of the NHS, the NIHR, or the Department of Health and Social Care. RW was supported by the Austrian Science Fund (FWF) [J4025-B26]. MC-O was funded by the EPSRC Tier-2 capital grant EP/P020259/1.

## Conflict of Interest

JB is a shareholder of Tailor Bio Ltd, Rutland, United Kingdom; receives honoraria from GlaxoSmithKline, London, United Kingdom and AstraZeneca, Cambridge, United Kingdom; receives research funding from Aprea Therapeutics AB, Massachusetts, United States; and holds patents for methods for predicting treatment response in cancers. ES receives honoraria from GlaxoSmithKline, London, United Kingdom and GE Healthcare, Illinois, United States, and is co-founder and shareholder of Lucida Medical Ltd, Cambridge, United Kingdom.

The remaining authors declare that the research was conducted in the absence of any commercial or financial relationships that could be construed as a potential conflict of interest.

## Publisher’s Note

All claims expressed in this article are solely those of the authors and do not necessarily represent those of their affiliated organizations, or those of the publisher, the editors and the reviewers. Any product that may be evaluated in this article, or claim that may be made by its manufacturer, is not guaranteed or endorsed by the publisher.
