# Supplementary material for: Clinically Interpretable Radiomics-Based Prediction of Histopathologic Response to Neoadjuvant Chemotherapy in High-Grade Serous Ovarian Carcinoma
Source: Front Oncol. 2022 Jun 16;12:868265. doi: 10.3389/fonc.2022.868265 (PMC9243357; doi:10.3389/fonc.2022.868265)
Supplement: Supplementary file 1 [file DataSheet_1.pdf]

Supplementary Materials

Clinically interpretable radiomics-based prediction of histopathologic response to neoadjuvant chemotherapy in high-grade serous ovarian carcinoma

Characteristics of Patient Cohorts

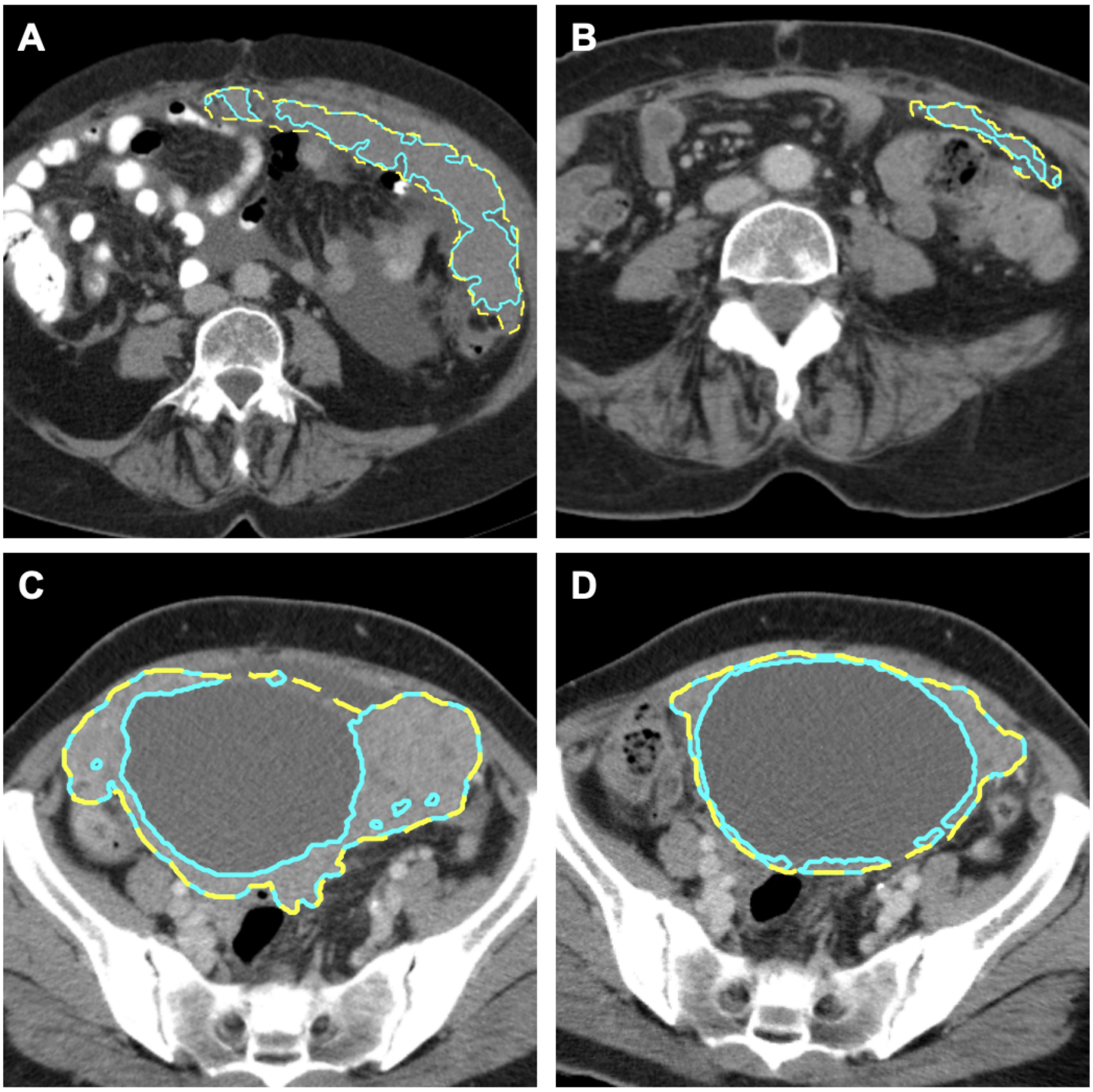

Figure S1: Examples of input CT images with the delineated omental lesions: **(A)** and **(B)** are the pre-NACT and pre-DPS CT scans, respectively, for a patient of the discovery dataset classified as CRS3; **(C)** and **(D)** are the pre-NACT and pre-DPS CT scans, respectively, for a patient of the external test dataset classified as CRS2 (incomplete response). The whole tumor and the solid/soft tissue (i.e., intermediately dense) components are represented by dashed yellow and solid cyan contours, respectively.

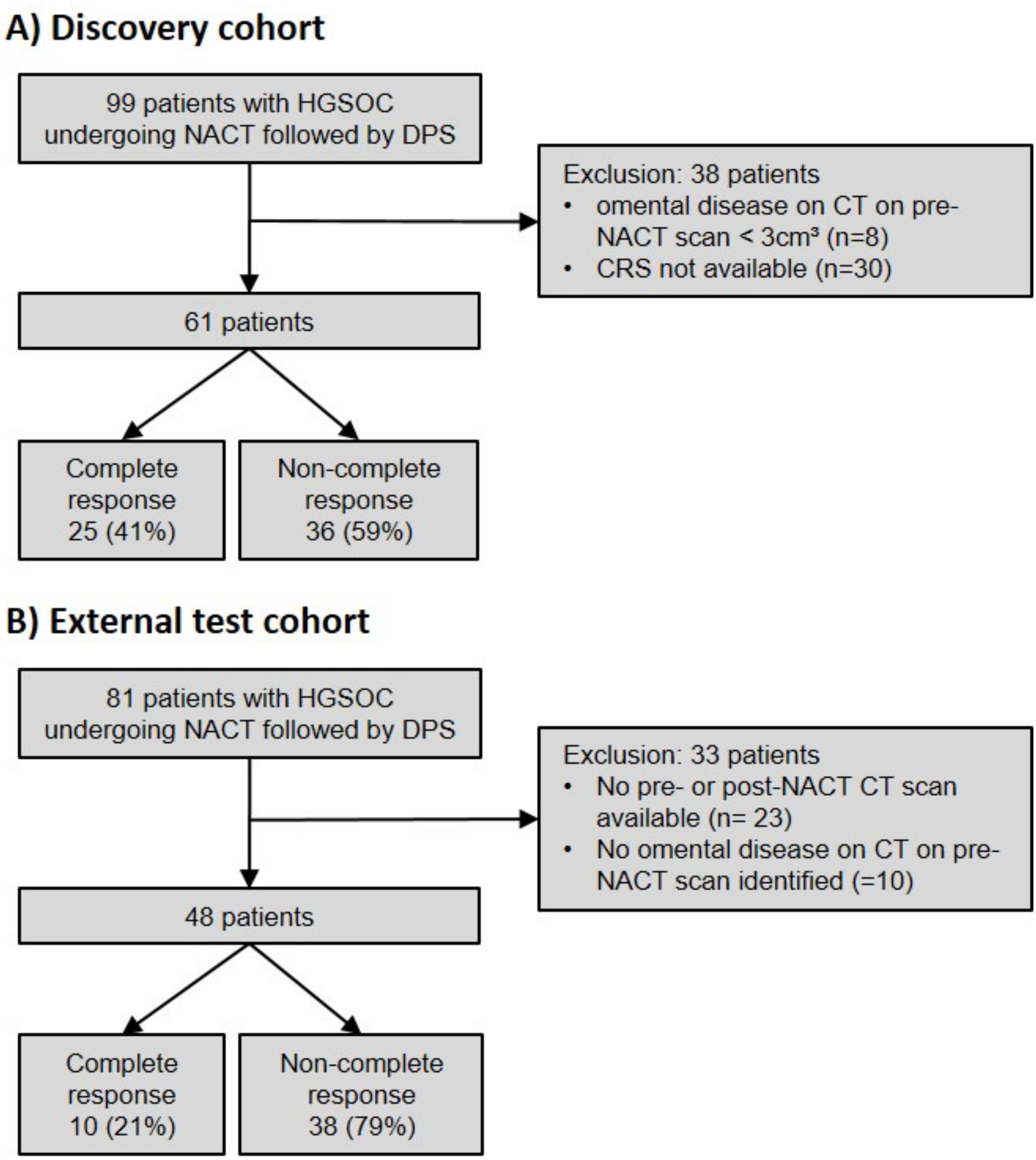

Figure S2: Study flowchart. Patients were included at the **(A)** Cambridge University Hospitals NHS Trust in the discovery set and **(B)** at the Barts Health NHS Trust in the external test set.

Training and testing methodology

The use of nested k-fold CV allows for model training where its hyperparameters also need to be optimized (1). The hyperparameter selection (λ in the case of the Elastic Net regularization) by means of non-nested CV could yield a biased model, leading to over-optimistic performance. With more details, the selection of a model without nested CV uses the same data to tune model hyperparameters and evaluate model performance. As a result, the model could be affected by overfitting on the training data with poor generalization capability (on 'unseen' data) (2,3).

The models were trained in the inner CV loop and selected according to the maximum Area Under the receiver operating characteristic Curve (AUC). To improve the estimated performance of the developed machine learning models, the fitting was repeated 100 times with different random permutations of the discovery dataset, thus resulting in 500 distinct models in CV. Performance metrics were averaged across these independent repetitions.

During the inner CV loop, the optimal operating point of the receiver operating characteristic (ROC) curve was estimated by using the slope s according to Equation (1):

$$s = \frac{Cost(P|N) - Cost(N|N)}{Cost(N|P) - Cost(P|P)} \cdot \frac{N}{P}, \tag{1}$$

where $Cost(N|P)$ and $Cost(P|N)$ are the costs of misclassifying a positive class as a negative class and a negative class as a positive class, respectively, while P and N denote the total numbers in the positive and negative class, respectively. Therefore, the optimal operating point is defined by the intersection of the straight line with slope s from the upper left corner of the ROC axes (False Positive Ratio = 0, True Positive Ratio = 1) and the ROC curve (4).

Volumetric Analyses

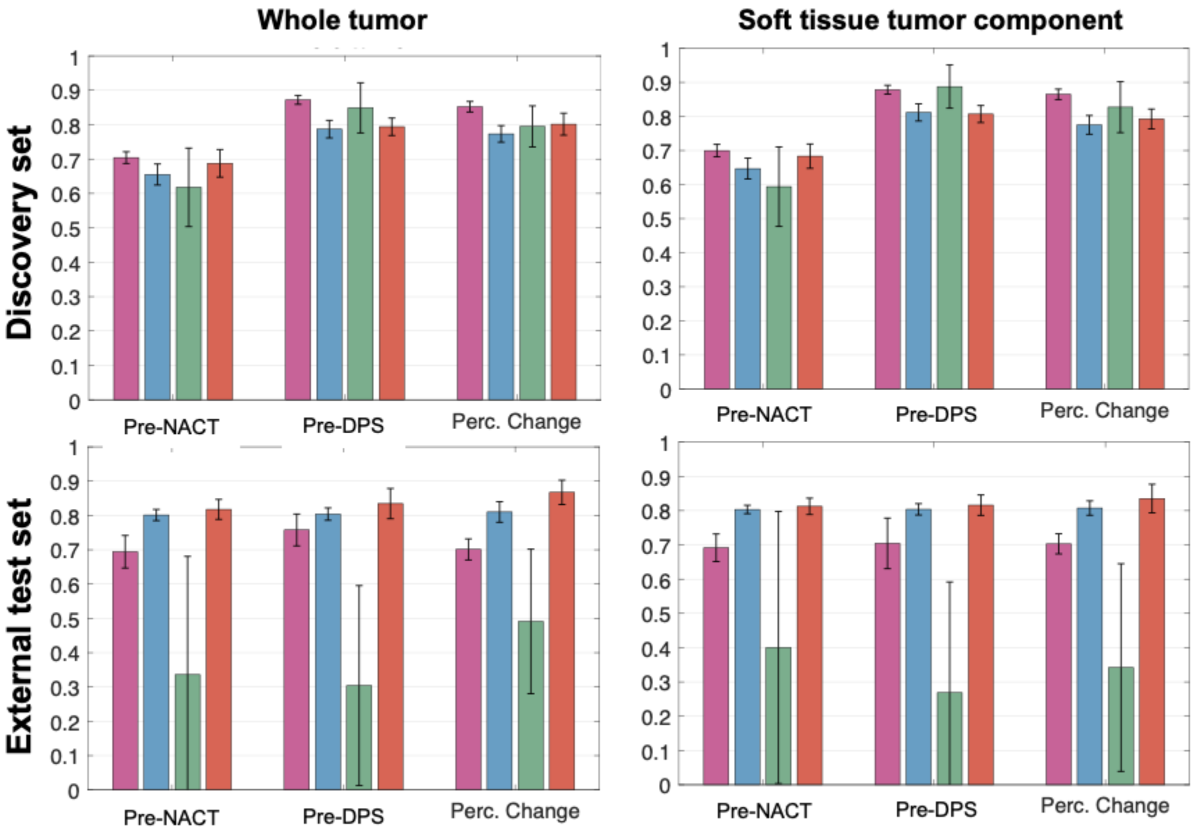

Figure S3. Comparison between the volume-based models based on the whole tumor and solid components.

Radiomics Analyses

Radiomic feature extraction, calibration and pre-processing

For the quantization, required in radiomic feature extraction, we used the Freedman-Diaconis rule, an extension of Scott's rule to non-Gaussian distributions, to find the optimal bin width of a distribution (i.e., histogram) for an unbiased estimation of the underlying probability density function. The Freedman-Diaconis rule is based on the interquartile range (IQR) and states that the optimal bin width of a distribution X can be defined as:

$$width_{bins} = 2\frac{IQR(X)}{N^{1/3}}, \quad (2)$$

where N is the number of voxels in the distribution X.

Considering a median bin number of 133.369 and 101.401 for whole tumor and solid tumor, respectively, we achieved a median bin width of 1.881 and 1.646. Therefore, the most suitable solution was a bin width of 2 for both whole and solid tumor VOIs.

The adopted pre-processing are listed in what follows:

- Intrinsic dependency analysis: to take into account the acquisition characteristics that might affect radiomic feature extraction, we calculated the Spearman correlation coefficient for each radiomic feature against each considered CT acquisition and reconstruction parameter, namely: (*i*) scanner vendor, (*ii*) scanner model, (*iii*) convolution kernel, (*iv*) KVP, (*v*) slice thickness, and (*vi*) pixel spacing. In particular, we considered $p<0.001$ (without multiple-comparison correction to keep a reasonable number of features) as a cut-off to discard the features correlated with the CT acquisition characteristics;
- Feature robustness analysis: Aiming at identifying the features robust against VOI variations (5), the ICC was considered to determine the most robust features extracted on whole tumor and solid/soft tissue tumor component VOIs obtained by an automated tissue-specific sub-segmentation method developed previously (6). Let k be the number of raters/measurements, The two-way random-effects model (or mixed-effects), consistency, single rater/measurement, $ICC(3,1)$ was used (7):

$$ICC(3,1) = \frac{MS_R - MS_E}{MS_R + (k-1)\, MS_E}, \quad (3)$$

  where MS_R and MS_E are the mean square for rows and mean square for error, respectively. The cut-off value θ ($\theta \in \{0.8, 0.9\}$) was optimized as a hyperparameter.
- Near-zero variance analysis was aimed at removing the features that do not convey information content (8). This operation considers a cut-off for the ratio of the most common value to the second most common value and a cut-off for the percentage of distinct values out of the number of total samples. We used the default values 95/5 and 10 for the two cut-offs, respectively.

Starting from the original 107 features (listed in **Table S4**) extracted by PyRadiomics, six features were found to be highly correlated with at least one CT acquisition parameter (in brackets):

- First Order: 90th Percentile (KVP)
- First Order: Median (KVP)
- GLCM: Inverse Difference (slice thickness & pixel spacing)
- GLCM: Inverse Difference Moment (slice thickness & pixel spacing)
- GLCM: Inverse Variance (pixel spacing)

- GLSZM: Large Area Low Gray Level Emphasis (slice thickness)

We performed a feature robustness analysis on the remaining 101 features. Features were deemed robust based on the ICC(3,1) – with θ=0.8 – between the radiomic features computed on the whole tumor against the solid tumor VOIs. This step was motivated by the experimental findings in **Fig. 4**. By doing so, 42 features were highly robust. Lastly, the near-zero variance analysis did not identify any feature to remove.

Elastic Net modeling and hyper-parameter optimization

The predictive modeling made use of the Elastic Net regularization for logistic regression with the dichotomized CRS as the response variable (9). Elastic Net uses a mixture between ℓ_1 and ℓ_2 regularization: the ℓ_1 regularization – also known as Least Absolute Shrinkage and Selection Operator (LASSO) (10,11) reduces the coefficients of certain features to zero, thus reducing the number of variables in a sparse model; the ℓ_2 penalty term – also called ridge regression (12) – constrains the magnitude of the feature coefficients so that a model is not dominated by any single feature. Let α be the weight for ℓ_1 and ℓ_2 penalties, also known as the mixing parameter.

As a hyperparameter tuning, we considered $\alpha \in \{0.10, 0.25, 0.50, 0.75, 0.90, 1.0\}$. A hyper-parameter optimization process was performed by considering 30 repetitions for each configuration (**Fig. S4**); even though the performance is generally robust against the hyper-parameter variations, the best configuration was provided by the pair <θ=0.8, α=0.9>.

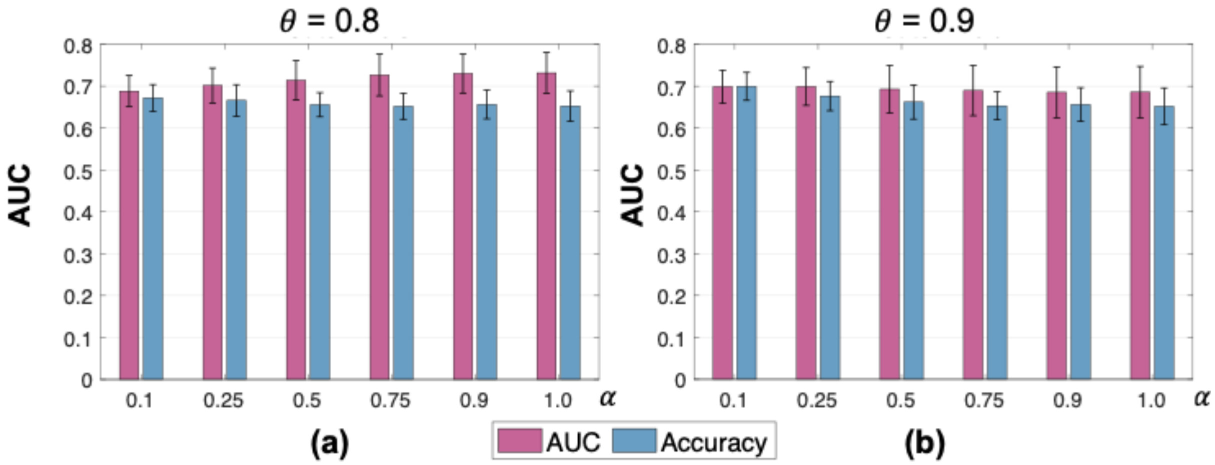

Figure S4: Hyper-parameter optimization of the radiomic models in terms of α (Elastic Net ℓ_1/ℓ_2 regularization parameter) and cut-off value θ=0.8 **(A)** and θ=0.9 **(B)**. The Elastic Net models were trained in 5-fold nested CV and the process was repeated 30 times for each configuration. The considered evaluation metrics were AUC and accuracy. The bar graph and error bars denote the average value and the standard deviation, respectively.

Post-processing and relevant feature analysis

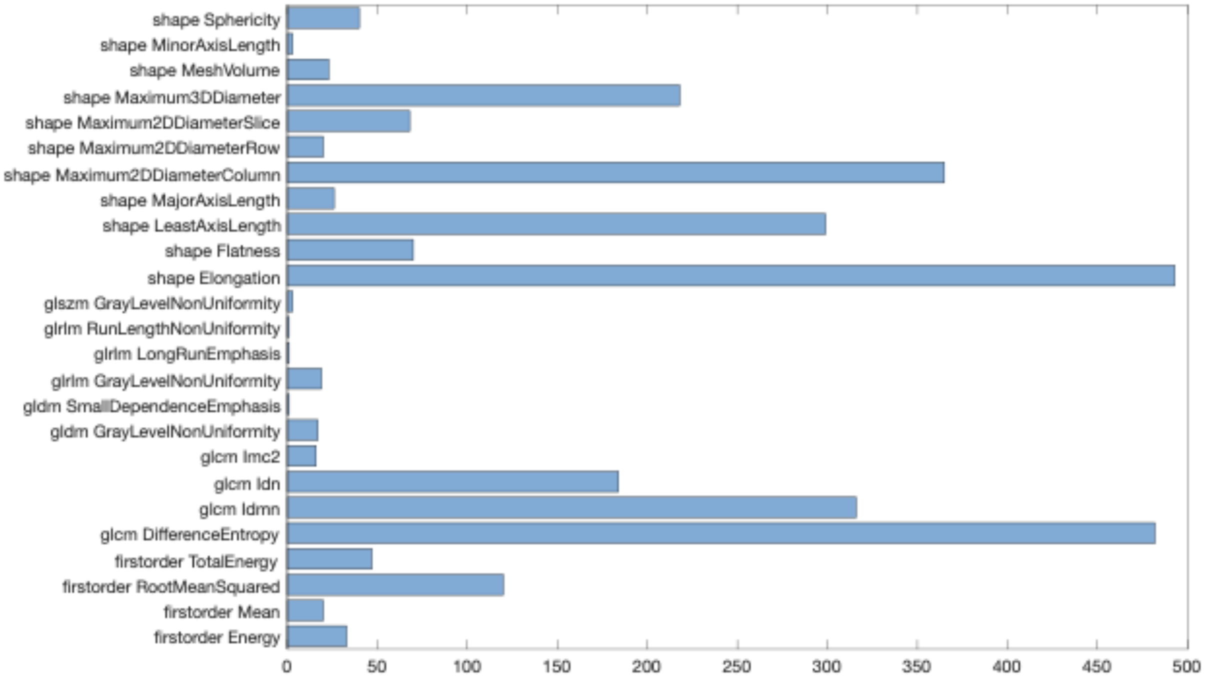

Figure S5: Relevant radiomic feature analysis of the Elastic Net models considering the features selected after 100 repetitions on the nested 5-fold CV on the discovery cohort. A total of 500 models were trained.

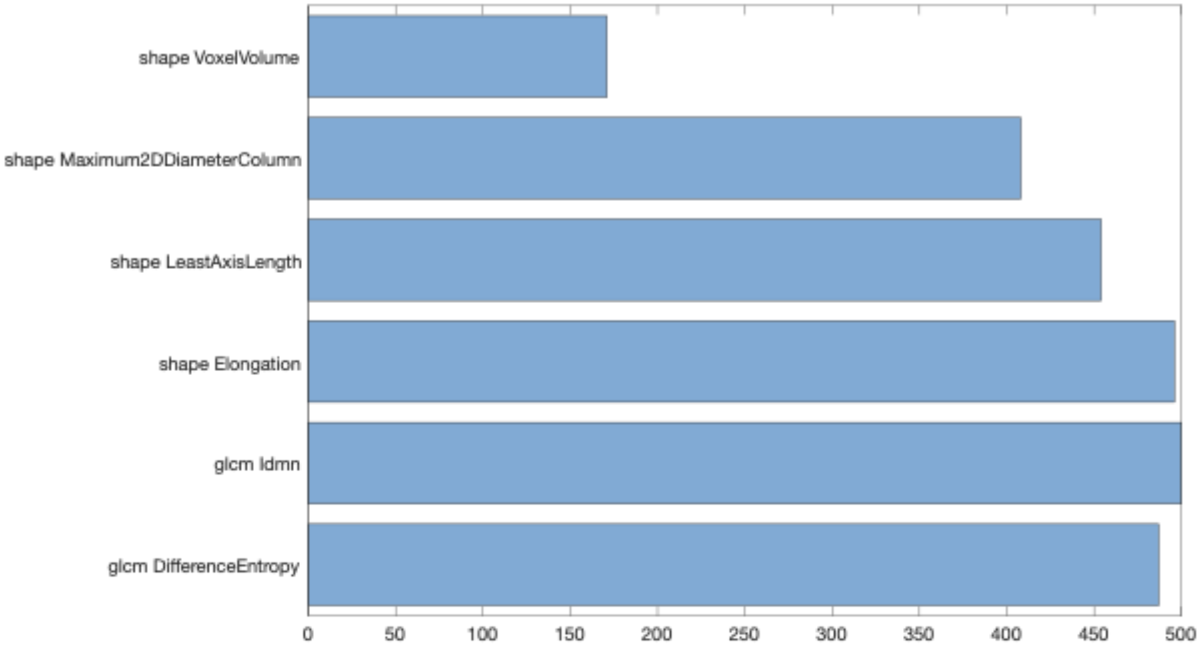

Figure S6: Relevant radiomic features of the Elastic Net models trained on the most-relevant feature subset considering the features selected after 100 repetitions on the nested 5-fold CV on the discovery cohort. A total of 500 models were trained.

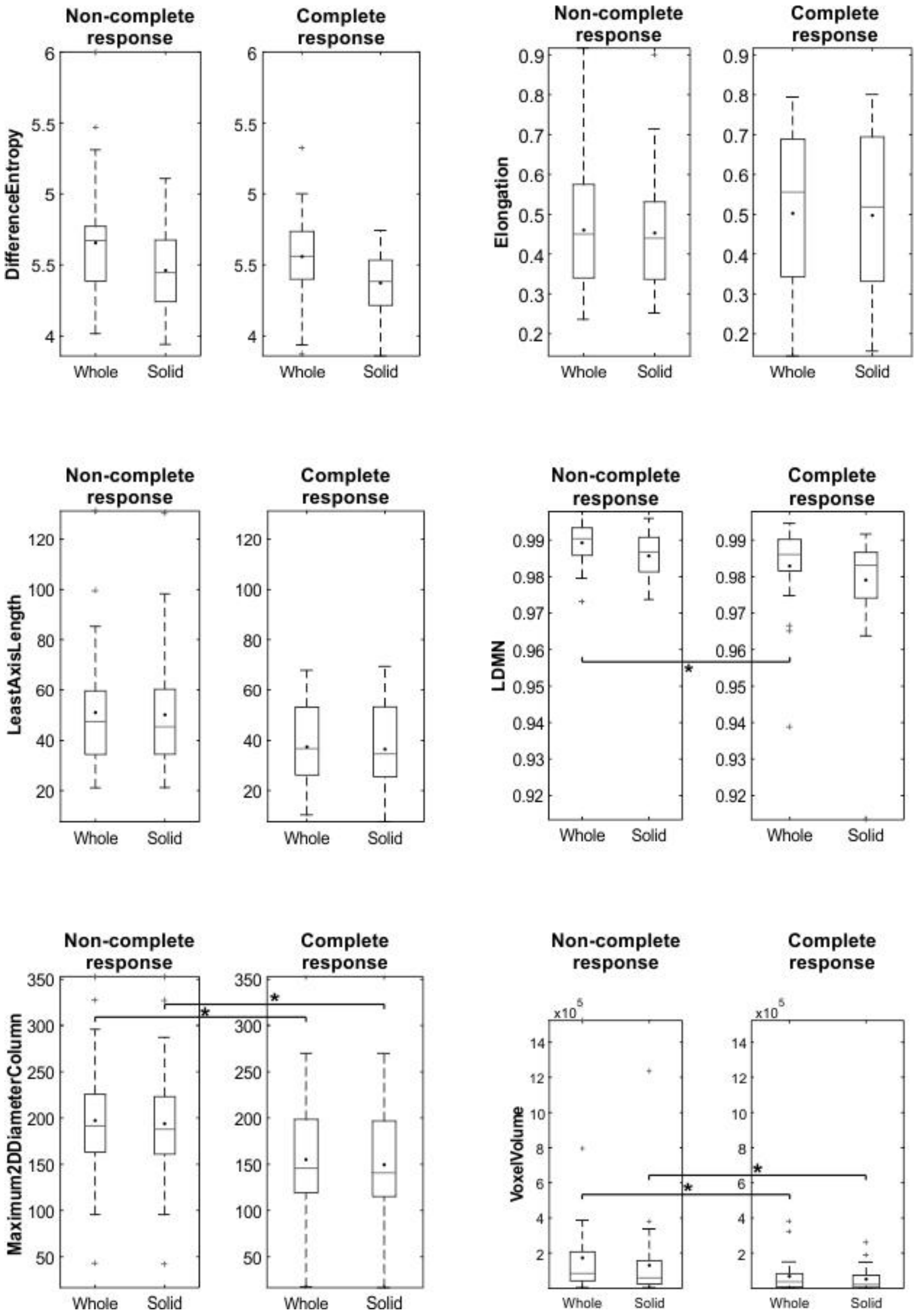
Non-complete response
Complete response
DifferenceEntropy
6
5.5
5
5.5
4
Whole
Solid
Elongation
0.9
0.8
0.7
0.6
0.5
0.4
0.3
0.2
LeastAxisLength
120
100
80
60
40
20
LDMN
0.99
0.98
0.97
0.96
0.95
0.94
0.93
0.92
*
Maximum2DDiameterColumn
350
300
250
200
150
100
50
VoxelVolume
x10^5
14
12
10
8
6
4
2

frontiers

Figure S7: Boxplots of the five most relevant radiomic features and volume, for the discovery cohort. Brackets indicate significant differences between patients with complete and non-complete response. Notation: * $p<0.05$.

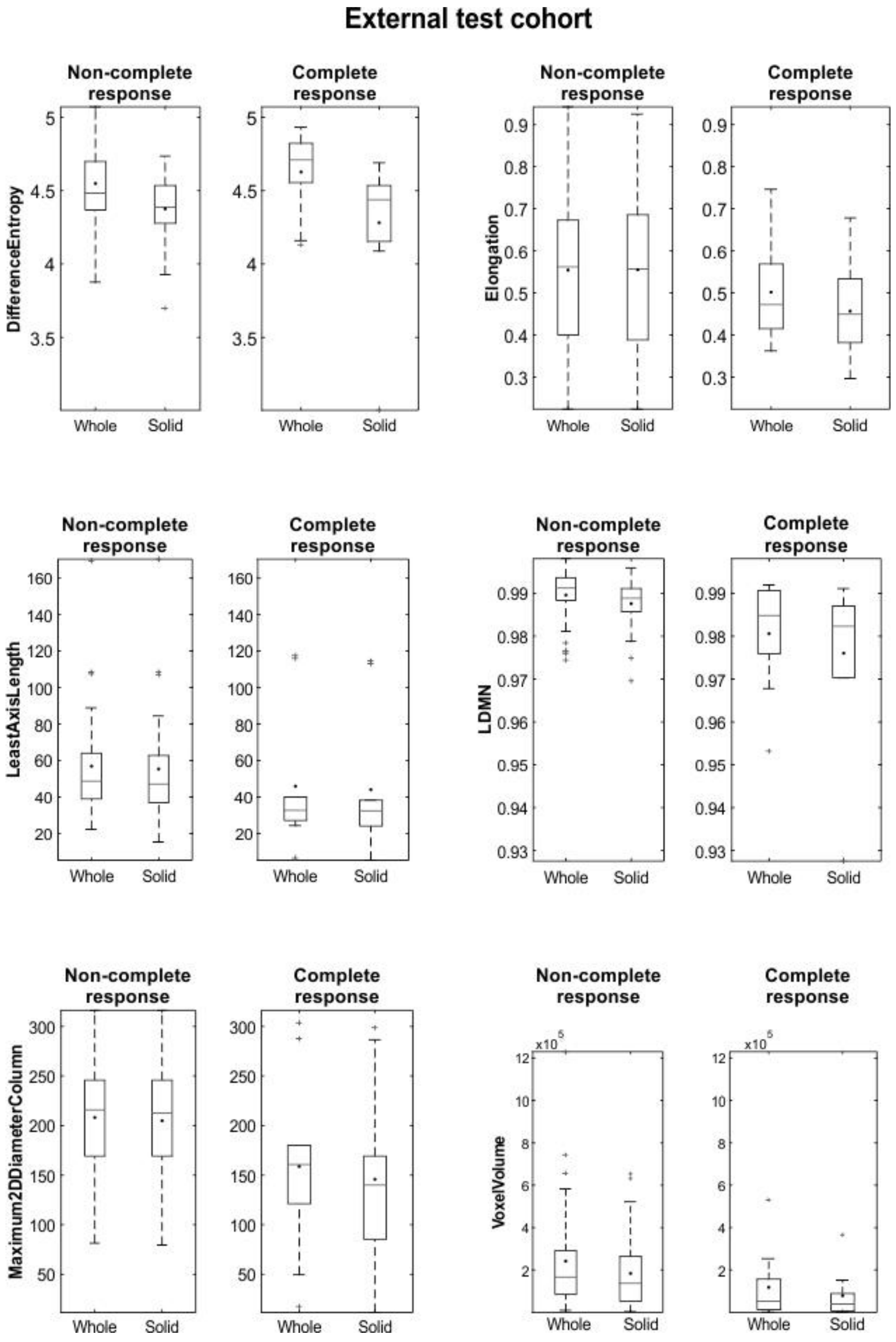

Figure S8: Boxplots of the five most relevant radiomic features and volume, for the external test cohort. No significant differences were found between patients with complete and non-complete response.

Patient Demographics

Table S1. Comparison of patient characteristics between patients with histopathologic response and response in patients with high grade serous ovarian cancer (**A**) in the discovery (NeOv) and (**B**) external testing (Barts) set. Data are given as absolute numbers and the proportion of patients, with the median and IQR, or mean ± standard deviation.

	**A**<br>**Discovery, n=61**			**B**<br>**External test, n=48**		
CRS	No response,<br>n = 36	Response,<br>n = 25	*p*-value	No response,<br>n=38	Response,<br>n=10	*p*-value
Age (years)	63± 12	62 ± 11	.70	62 ± 12	64 ± 13	.76
FIGO			.27			.25
IIIC	27 (75%)	15 (60%)		27 (71%)	8 (80%)	
IV	9 (25%)	10 (40%)		11(29%)	3 (20%)	
CA125 pre-therapy, U/mL	950 (1635)	1693 (3158)	.31	899 (1451)	1663 (2046)	.58
Omental tumor volume pre-therapy, cm^3	85 (170)	37 (84)	.007	167 (209)	65(194)	0.005
BRCA germline mutation			.51			.74
Unknown	11 (31%)	4 (16%)		34 (87%)	8 (80%)	
BRCA1	4 (11%)	4 (16$)		3 (8%)	2 (20%)	
BRCA2	2 (6%)	3 (12%)		1 (3%)	0 (0%)	

Wild type	19 (52%)	14 (56%)		0 (0%)	0 (0%)	
Number of NACT cycle			.33			.51
3	25 (69%)	18 (72%)		24 (64%)	8 (80%)	
>3	11 (31%)	7 (28%)		14 (36%)	2 (20%)	
Outcome of IDS			.008			.45
No residual disease	13 (36%)	20 (80%)		28 (74%)	9 (90%)	
≤1cm	15 (42%)	4 (16%)		5 (13%)	1 (10%)	
>1cm	8 (24%)	1 (4%)		5 (13%)	0 (0%)	

IDS, interval debulking surgery; NACT, neoadjuvant chemotherapy; FIGO, Fédération Internationale de Gynécologie et d'Obstétrique.

Table S2. Univariable analysis of the influence of clinical and radiological variables on progression-free survival in patients with high grade serous ovarian cancer in the (**A**) discovery and (**B**) external test set.

	**A** **Discovery, n=61**			**B** **External test, n=48**		
	HR	95% CI	*p*-value	HR	95% CI	*p*-value
Noncomplete response	2.0	1.15-3.47	.01	2.23	0.98-5.01	.057
Age (years)	1.01	0.99-1.04	.44	1.01	0.98-1.04	.59
FIGO (IIC vs. IV)	1.16	0.66-2.05	.60	0.77	0.37-1.63	.45

Pre-treatment CA125 (per U/ml)	1.0	1.0-1.0	.54	1.0	1.0-1,0	.37
NACT cycles (per cycle)	1.06	0.85-1.33	.59	1.13	0.76-1.69	.54
Outcome of DPS (>1cm vs. NRD)	3.47	1.51-7.94	.003	2.89	0.99-8.45	.053
Omental tumor volume pre-therapy (per cm³)	1.0	1.0-1.0	.50	1.0	1.0-1.0	.13
HR, hazard ratio; CI, confidence interval; NA, at applicable; NRD, no residual disease						

Table S3. Univariable analysis of the influence of clinical and radiological variables on overall survival in patients with high grade serous ovarian cancer in the (**A**) discovery and (**B**) external test set.

	**A** Discovery, n=61			**B** External test, n=49		
	HR	95% CI	*p*-value	HR	95% CI	*p*-value
No pathologic response	1.71	0.96-3.06	.07	2.97	0.89-9.92	.08
Age (years)	1.0	0.98-1.03	.79	1.01	0.97-1.04	.81
FIGO (IIC vs. IV)	0.79	0.43-1.45	.44	1.55	0.66-3.66	.31
Pre-treatment CA125 (per U/ml)	1.0	1.0-1.0	.44	1.0	1.0-1,0	.53
NACT cycles (per cycle)	0.99	0.78-1.25	.90	1.05	0.64-1.71	.85
Outcome of DPS (>1cm vs. NRD)	1.8	0.78.4.28	.17	7.56	2.61-21.91	<.001

Omental tumor volume pre-therapy (per cm³)	1.0	1.0-1.0	.79	1.0	1.0-1.0	.01
HR, hazard ratio; CI, confidence interval; NRD, no residual disease						

CT Imaging Acquisition and Radiomic Feature Extraction

Table S4. CT acquisition and reconstruction parameters for the development and external test datasets.

Parameter	Discovery dataset	External test dataset
Scanner vendor	GE, Siemens, Toshiba	GE, Siemens, Toshiba, Philips
Matrix size (pixels)	512×512	512×512
Pixel spacing (mm)	0.53-0.93 (mean 0.70)	0.61-0.95 (mean 0.77)
Slice thickness (mm)	2.0-5.0	3.0,5.0
Reconstruction kernel	Multiple	Multiple
KVP	100, 120, 130, 140	100, 120

Table S5. Radiomic features extracted from the VOIs in this study. All radiomic features were extracted using PyRadiomics and the radiomic feature formulation can be found on the online PyRadiomics documentation (https://pyradiomics.readthedocs.io/en/latest/)

#	Radiomic feature
First-order	
1	10th Percentile
2	90th Percentile
3	Energy
4	Entropy
5	Interquartile Range
6	Kurtosis
7	Maximum

8	Mean Absolute Deviation
9	Mean
10	Median
11	Minimum
12	Range
13	Robust Mean Absolute Deviation
14	Root Mean Squared
15	Skewness
16	Total Energy
17	Uniformity
18	Variance
Shape-based (3D)	
19	Mesh Volume
20	Voxel Volume
21	Surface Area
22	Surface Area to Volume ratio
23	Sphericity
24	Maximum 3D diameter
25	Maximum 2D diameter (slice)
26	Maximum 2D diameter (column)
27	Maximum 2D diameter (row)

28	Major Axis Length
29	Minor Axis Length
30	Least Axis Length
31	Elongation
32	Flatness
Gray Level Co-occurrence Matrix (GLCM)	
33	Autocorrelation
34	Cluster Prominence
35	Cluster Shade
36	Cluster Tendency
37	Contrast
38	Correlation
39	Difference Average
40	Difference Entropy
41	Difference Variance
42	ID: Inverse Difference
43	IDM: Inverse Difference Moment
44	IDMN: Inverse Difference Moment Normalized
45	IDN: Inverse Difference Normalized
46	IMC 1: Informational Measure of Correlation 1
47	IMC 2: Informational Measure of Correlation 2

48	Inverse Variance
49	Joint Average
50	Joint Energy
51	Joint Entropy
52	MCC: Maximal Correlation Coefficient
53	Maximum Probability
54	Sum Average
55	Sum Entropy
56	Sum Squares
Gray Level Dependence Matrix (GLDM)	
57	Dependence Entropy
58	Dependence NonUniformity
59	Dependence NonUniformity Normalised
60	Dependence Variance
61	Gray Level NonUniformity
62	Gray Level Variance
63	High Gray Level Emphasis
64	Large Dependence Emphasis
65	Large Dependence High Gray Level Emphasis
66	Large Dependence Low Gray Level Emphasis
67	Low Gray Level Emphasis

68	Small Dependence Emphasis
69	Small Dependence High Gray Level Emphasis
70	Small Dependence Low Gray Level Emphasis
Gray Level Run Length Matrix (GLRLM)	
71	Gray Level NonUniformity
72	Gray Level NonUniformity Normalised
73	Gray Level Variance
74	High Gray Level Run Emphasis
75	Long Run Emphasis
76	Long Run High Gray Level Emphasis
77	Long Run Low Gray Level Emphasis
78	Low Gray Level Run Emphasis
79	Run Entropy
80	Run Length NonUniformity
81	Run Length NonUniformity Normalised
82	Run Percentage
83	Run Variance
84	Short Run Emphasis
85	Short Run High Gray Level Emphasis
86	Short Run Low Gray Level Emphasis
Gray Level Size Zone Matrix (GLSZM)	

87	Gray Level NonUniformity
88	Gray Level NonUniformity Normalised
89	Gray Level Variance
90	High Gray Level Zone Emphasis
91	Large Area Emphasis
92	Large Area High Gray Level Emphasis
93	Large Area Low Gray Level Emphasis
94	Low Gray Level Zone Emphasis
95	Size Zone NonUniformity
96	Size Zone NonUniformity Normalised
97	Small Area Emphasis
98	Small Area High Gray Level Emphasis
99	Small Area Low Gray Level Emphasis
100	Zone Entropy
101	Zone Percentage
102	Zone Variance
Neighboring Gray-Tone Difference Matrix (NGTDM)	
103	Busyness
104	Coarseness
105	Complexity
106	Contrast

107	Strength

References

1. Parvandeh S, Yeh H-W, Paulus MP, McKinney BA. Consensus features nested cross-validation. Bioinformatics. 2020;36:3093–8.

2. Cawley GC. Over-Fitting in Model Selection and Its Avoidance [Internet]. Advances in Intelligent Data Analysis XI. 2012. page 1–1. Available from: http://dx.doi.org/10.1007/978-3-642-34156-4_1

3. Doran SJ, Kumar S, Orton M, d'Arcy J, Kwaks F, O'Flynn E, et al. "Real-world" radiomics from multi-vendor MRI: an original retrospective study on the prediction of nodal status and disease survival in breast cancer, as an exemplar to promote discussion of the wider issues. Cancer Imaging. 2021;21:37.

4. Briggs WM, Zaretzki R. The Skill Plot: a graphical technique for evaluating continuous diagnostic tests. Biometrics. 2008;64:250–6; discussion 256–61.

5. Le EPV, Rundo L, Tarkin JM, Evans NR, Chowdhury MM, Coughlin PA, et al. Assessing robustness of carotid artery CT angiography radiomics in the identification of culprit lesions in cerebrovascular events. Sci Rep. 2021;11:3499.

6. Rundo L, Beer L, Ursprung S, Martin-Gonzalez P, Markowetz F, Brenton JD, et al. Tissue-specific and interpretable sub-segmentation of whole tumour burden on CT images by unsupervised fuzzy clustering. Comput Biol Med. 2020;120:103751.

7. McGraw KO, Wong SP. Forming inferences about some intraclass correlation coefficients [Internet]. Psychological Methods. 1996. page 30–46. Available from: http://dx.doi.org/10.1037/1082-989x.1.1.30

8. Papanikolaou N, Matos C, Koh DM. How to develop a meaningful radiomic signature for clinical use in oncologic patients. Cancer Imaging. 2020;20:33.

9. Zou H, Hastie T. Regularization and variable selection via the elastic net [Internet]. Journal of the Royal Statistical Society: Series B (Statistical Methodology). 2005. page 301–20. Available from: http://dx.doi.org/10.1111/j.1467-9868.2005.00503.x

10. Tibshirani R. Regression shrinkage and selection via the lasso: a retrospective [Internet]. Journal of the Royal Statistical Society: Series B (Statistical Methodology). 2011. page 273–82. Available from: http://dx.doi.org/10.1111/j.1467-9868.2011.00771.x

11. Cai J, Zheng J, Shen J, Yuan Z, Xie M, Gao M, et al. A Radiomics Model for Predicting the Response to Bevacizumab in Brain Necrosis after Radiotherapy. Clin Cancer Res. 2020;26:5438–47.

12. Hoerl AE, Kennard RW. Ridge Regression: Biased Estimation for Nonorthogonal Problems [Internet]. Technometrics. 2000. page 80–6. Available from: http://dx.doi.org/10.1080/00401706.2000.10485983
